# Aprepitant, a Neurokinin-1 Receptor Antagonist, as a Disruptive Drug for the Treatment of Pediatric Cancer

**DOI:** 10.3390/jcm15187004

**Published:** 2026-09-10

**Authors:** Marisa Rosso, Carlos Alcaide, Miguel Muñoz

**Affiliations:** 1 Research Laboratory on Neuropeptides, Institute of Biomedicine of Seville (IBIS), 41013 Seville, Spain; marisarossog@gmail.com; 2 Department of Pediatric Oncology, Hospital Regional Universitario de Málaga, 29010 Malaga, Spain; carlosalcaidealvarez@gmail.com

**Keywords:** pediatric cancer, antitumor, apoptosis, antimetastasis, substance P, neurokinin-1 receptor, neurokinin-1 receptor antagonist

## Abstract

Although advances in pediatric oncology have resulted in cure rates exceeding 80% among children with cancer, progress in survival outcomes has begun to plateau in recent years. Mortality in this population remains largely associated with aggressive disease features, particularly tumor resistance to chemotherapy and metastatic spread. At the same time, the growing population of childhood cancer survivors has drawn increasing attention to the persistent and potentially serious late effects associated with conventional chemotherapy. Thus, it is crucial to discover drugs with specific antitumor action, effective and safe drugs that, combined with chemotherapy or radiotherapy, could chemosensitize or radiosensitize the tumor and reduce the severe side effects of both. Substance P (SP) peptide and its Neurokinin-1 receptor (NK-1R) are known to be involved in pediatric cancer, promotion and progression. Pediatric cancer overexpresses SP/NK-1R and NK-1R is essential for viability of cancer cells and is not essential for normal non-tumor cells. SP induces mitogenesis, exerts anti-apoptotic effects, and promotes angiogenesis, invasion and migration for metastasis in cancer cells and produces inflammation. Conversely, NK-1R antagonists, such as aprepitant and similar drugs, inhibit mitogenesis and induce apoptosis in pediatric cancer cells in a concentration-dependent manner. They also inhibit angiogenesis, invasion, and migration in pediatric cancer cells and have an anti-inflammatory effect. Moreover, aprepitant in combination with chemotherapy or radiotherapy can produce chemosensitization or radiosensitisation and decreases the severe side effects of both. This review updates the role of the SP/NK-1R axis in pediatric cancers and analyzes its potential as a therapeutic target. It highlights NK-1R antagonists, particularly aprepitant, and their potential as drugs for the treatment of pediatric cancer.

## 1. Introduction

Over the past five decades, advances in pediatric oncology have markedly improved survival outcomes for children with cancer, with current estimates indicating that more than 80% of affected children can now achieve long-term survival [1,2]. Nevertheless, the pace of progress has declined in recent years, suggesting that conventional cytotoxic chemotherapy may have reached important therapeutic limitations. Moreover, the growing population of childhood cancer survivors has highlighted the considerable long-term toxicities associated with chemotherapy, emphasizing the need for safer and more effective therapeutic approaches [3]. The reasons for the incurability of these tumors are varied: chemoresistance, the development of paracrine mechanisms in their microenvironment, immune evasion, or the development of pro-tumor inflammatory mechanisms, among others [4]. Among the causes of death in pediatric cancer, those attributable to tumor chemoresistance and metastasis should be highlighted. Resistance to chemotherapy remains a major challenge in cancer management, as it can markedly compromise the effectiveness of anticancer treatment. Furthermore, metastatic dissemination is a critical determinant of cancer mortality and is estimated to be responsible for approximately 90% of cancer-related deaths [5]. Furthermore, conventional chemotherapy remains the backbone of treatment despite a narrow therapeutic index and substantial acute and late toxicity, even though it produces frequent adverse effects such as chemotherapy-induced nausea and vomiting (CINV), febrile neutropenia, neutropenic enterocolitis, cardiotoxicity, hepatotoxicity, nephrotoxicity, and neurotoxicity, and some of these effects pose a risk to the lives of these patients, while others can leave permanent sequelae. Therefore, it is crucial to discover safe and effective new drugs (with specific antitumor action) that, in combination therapy with chemotherapy or radiotherapy, can (a) chemosensitize or radiosensitize the tumor and (b) reduce the severe toxicity of chemotherapy.

The overarching objective of this approach is to enhance treatment outcomes in children with cancer while preserving quality of life and minimizing long-term treatment-related toxicity. Chemotherapy-induced nausea and vomiting (CINV) is largely associated with activation of the neurokinin-1 receptor (NK-1R) by its endogenous ligand, substance P (SP). Notably, NK-1R is highly expressed in brain regions involved in the emetic response, particularly the area postrema and the nucleus of the solitary tract, which play important roles in the initiation and regulation of nausea and vomiting [6]. This important finding led to the discovery of the first NK-1R antagonist drug for CINV, called aprepitant, which acts centrally on NK-1Rs in vomiting centers within the central nervous system (CNS) to block their activation by SP released as an unwanted consequence of chemotherapy [7]. In addition, it is known that SP and NK-1R are involved in neurogenic inflammation [8] and cancer promotion and progression [9].

SP increases tumor cell proliferation in a concentration-dependent manner and has an anti-apoptotic effect on tumor cells, increases angiogenesis and produces invasion and migration of tumor cells (metastasis). Tumor cells overexpress SP and NK-1R [9] compared to normal non-tumor cells. In contrast, NK-1R antagonists block the actions mediated by SP in neurogenic inflammation and cancer; these antagonists show both anti-inflammatory and antitumor activity: NK-1R antagonists inhibit mitogenesis and promote apoptosis in tumor cells and also block angiogenesis, the migration and invasion of cancer cells [8,10,11,12,13,14,15,16,17,18]. In addition, aprepitant, an NK-1R antagonist, is a broad-spectrum anticancer drug with action against pediatric cancer [19]. Furthermore, these NK-1R antagonists exert additional therapeutic effects, such as anti-CINV, antidepressant, hepatoprotective, neuroprotective, nephroprotective and cardioprotective actions [6,20,21,22,23,24]; they are safe and well tolerated [20]. Regarding safety, NK-1R antagonists differ from chemotherapy in this way, because chemotherapy (at therapeutic doses) exerts severe side effects; however, NK-1R antagonists, such as aprepitant (at therapeutic doses), are safe, and in addition, exert additional therapeutic effects. Moreover, the NK-1R antagonist aprepitant in combination therapy with chemotherapy or radiotherapy produces chemosensitization and radiosensitization in cancer cells and counteracts the severe side effects of both [25,26].

Herein, we review the involvement of SP/NK-1R in pediatric cancer and the antitumor activity of NK-1R antagonists as broad-spectrum anticancer drugs, and the use of these antagonists for the treatment of pediatric cancer is also suggested. In addition, studies are described that highlight the safety and ability of the NK-1R antagonist to improve antitumor activity in combination therapy with chemotherapy or radiotherapy, as well as to mitigate the serious side effects of both. We also evaluate whether the possible repurposing of already-marketed NK-1R antagonists, such as aprepitant, could be useful in developing new treatment strategies to overcome cancer resistance.

## 2. SP and NK-1R

SP is a neuropeptide that belongs to the tachykinin peptide family (Neurokinin A, Neurokinin B, and Hemokinin-1 (HK-1)) [27]. SP is classically known because it is in the CNS and in the peripheral nervous system (PNS); hence, it is classified as a neuropeptide: a short chain of amino acids (typically 3–100 residues) acting as neurotransmitter, neuromodulator or neurohormone. SP is present throughout the body: tissues, cells, and body fluids (blood, cerebrospinal fluid, saliva, etc.) [27]. SP is overexpressed in cancer tissues and cancer cells studied [9], and cancer patients have higher serum SP levels than normal subjects [28,29,30,31]. SP and NK-1R are also in inflammatory cells: neutrophils, eosinophils, T and B lymphocytes, monocytes, macrophages [8] and endothelial cells [32].

It is known that SP can be hydrolyzed by the following enzymes: (a) neutral endopeptidase (NEP), also known as membrane metallo-endopeptidase (MME), cluster of differentiation 10 (CD10) or common acute lymphoblastic leukemia antigen (CALLA), and (b) angiotensin-converting enzyme (ACE), also referred to as CD143, kinase II, or dipeptidyl carboxypeptidase. NEP is completely inhibited by phosphoramidon (an NEP inhibitor), and ACE by captopril (an ACE inhibitor) [33].

There are three main classes of neurokinin receptors—NK-1R, NK-2R and NK-3R— which mediate the biological effects of tachykinins SP, HK-1, neurokinin A, and neurokinin B. NK-1R mediates the biological activities of SP, which exhibits greater agonist potency than other tachykinins such as neurokinin A or neurokinin B. NK-1R has two isoforms: a full-length (NK-1R-FL) isoform of 407 amino acids and a truncated (NK-1R-T) isoform (of 311 amino acids, since 96 residues are lost at the C-terminus) [34]. These isoforms can initiate various intracellular signaling pathways and play different roles in both physiological and pathophysiological processes [35]. The NK-1R-FL isoform has a tenfold greater binding affinity for SP than the NK-1R-T isoform [36,37]. Cancer cells overexpress NK-1R isoforms [38] (Table 1), which are involved in the viability of these cells, whereas NK-1R is not involved in the viability of normal non-tumor cells [39]. NK-1R is in inflammatory cells: neutrophils, eosinophils, mast cells, T and B lymphocytes, monocytes, macrophages [8] and endothelial cells and platelets [32,40]. Furthermore, the NK-1R-T isoform is overexpressed in tumor samples and cancer cells [14,41,42]. Because SP is the naturally occurring ligand with the highest affinity for NK-1R, its biological action is primarily mediated by this receptor.

NK-1R is a G protein-coupled receptor, also known as a seven-transmembrane domain (7TM) receptor, that couples to a subset of G proteins. In addition, NK-1R induces the activation of nuclear factor kappa beta (NF-κB), which is involved in the control of the expression of proinflammatory cytokines [34,43,44]. In human astrocytoma cells, stimulation of the NK-1R receptor by SP or other NK-1R agonists triggers a wide range of cellular responses. These include the activation of phospholipase D, increased production of inositol phosphate, and the release of mediators such as interleukin-6 (IL-6), interleukin-8 (IL-8), taurine, and glutamate. NK-1R activation also promotes intracellular calcium mobilization, modulates glutamate and potassium transport, and enhances glycogen breakdown [43,44]. When NK-1R is stimulated, it activates secondary messengers that trigger various effector mechanisms that regulate cellular excitability and function, activating three second messenger systems: (a) stimulation, via phospholipase C, by phosphatidylinositol turnover, leading to both intracellular and extracellular calcium mobilization; (b) mobilization of arachidonic acid by phospholipase A2; and (c) cAMP accumulation by adenylate cyclase [43,44].

**Table 1:** Tumors and cancer cell lines. Expression of SP, NK-1R in cancer cells and tumor samples. Isoforms of NK-1R. The *TACR1* gene is essential for cancer cell viability. SP induces mitogenesis and the IC_50_ and IC_100_ of aprepitant for cancer cell lines.

Tumor	Cell Line	Aprepitant		SP nM	*TACR1* Gene Is Essential for Cell Viability	Isoforms NK-1R kDa	Presence of SP/NK-1R in Tumor Samples	
		IC_50_ μM	IC_100_ μM				SP	NK-1R
Neuroblastoma [10,19,41,42]	SKN-BE	24.6	48.8	100	Not studied	54	+by PCR-RT	*tr-TACR1* overexpress
	IMR-32	19.6	45.1	5		75, 54, 33		
	KELLY	27.7	49.5	10		54, 33		
Glioma [10,19,39,45,46]	GAMG	33.1	66.2	50	Yes	54, 38, 33	Not studied	+
	U87-MG	77		5–500		58, 33		
Cranio- Pharyngioma [47]	Not studied	Not studied	Not studied	Not studied	Not studied	Not studied	+	+
Hepatoblastoma [14]	HuH6	33.18		50	Not studied	Truncated isoforms overexpress	+	+
	HepT1	31.1		10				
	HepG2	38.61		50				
Retinoblastoma [11,12,19]	Y-79	30.4	59	100	Not studied	75, 58, 33	+	+
	WERIRb1	23	53.1	100		75, 58, 33		
Osteosarcoma [15]	MG-63	28.6	80	500	Not studied	46	Not studied	+
Rhabdoid Tumors [41]	BT-12	50	>100	200	Not studied	Truncated isoforms overexpress	+by PCR-RT	+by PCR-RT
	CHLA266	40	>100	200				
	G-401	50	>100	200				
T cells acute lymphoblastic leukemia [13,41]	BE-13	19.5	50	200	Yes	58, 33	+by PCR-RT	+by PCR-RT
B cells acute lymphoblastic leukemia [13,41]	SD-1	29.4	59.2	200	Yes	75, 58, 33	+by PCR-RT	+by PCR-RT
Acute myeloid leukemia [17,41,48]	KG-1	18.8	60	50–100	Not studied	60, 50	+by PCR-RT	+by PCR-RT
	HL-60	14.8	60					
	K562	46						
No Tumor	Cell line							
Human Embryonic Kidney [14,19]	HEK 293		>90		Not studied	Full-length isoforms overexpress		
Fibroblast [14]	Healthy patients	57.5			No	Not overexpress		
Lymphocyte [17]	Healthy donors	176.2	344.7		Not studied	Not overexpress		

## 3. SP/NK-1R in Pediatric Cancer

### 3.1. Neuroblastoma

Neuroblastoma (NB) is an embryonal malignancy arising from the aberrant growth of neural crest progenitor cells of the sympathetic nervous system. It is the most frequent cancer diagnosed during the first year of life and accounts for ~10% of pediatric cancers. NB is an embryonic neoplasm derived from the neural crest and the most common extracranial tumor in children, with highly heterogeneous clinical behavior. Low-risk patients have a 95% disease-free survival rate, compared with a 50% risk of relapse in high-risk patients. Key genetic features include MYCN amplification, 17q gains, 1p and 11q losses, and distal 6q deletions as a marker of poor prognosis [49]. Various models agree that NB is an immunologically “cold” tumor, characterized by a robust but inefficient inflammatory response to soluble signals from the tumor microenvironment (TME) such as neuropeptides, chemokines, ligands for the natural killer cell receptor (NKG2D), and senescence-associated secretory phenotype (SASP) products, which govern the plasticity between two major cell populations, one with noradrenergic and adrenergic differentiation (ADRN) and another with mesenchymal differentiation (MES); the latter cause the formation of premetastatic niches (PMNs), which is pivotal in promoting the spread of cancer cells, and therapeutic resistance via Therapy-Induced Senescence (TIS) [50,51]. The intensity of the treatments can transform this ADRN phenotype into a senescent MES phenotype, resistant to chemotherapy and exhibiting a strong SASP phenotype capable of establishing a developed epithelial–mesenchymal transition (EMT) in neighboring cells, remodeling the TME responsible for the invasion of structures, whereas relieving therapeutic (selective) pressure could favor a more proliferative, differentiated ADRN phenotype.

Furthermore, it has been reported that SKN-BE (2), IMR-32 and KELLY human NB cell lines express NK-1R isoforms and that IMR5, SKN-BE, SKN-AS, SY5Y and KELLY human NB cell lines express NK-1R protein and the tachykinin receptor 1 (*TACR1*) gene [10,16] (Table 1). Bone marrow involvement is detected at diagnosis in more than 90% of patients with high-risk neuroblastoma (NB) [52]. Notably, SP is present in approximately 90% of metastatic NB cells located in the bone marrow [53]. Experimental evidence indicates that SP promotes NB cell growth, whereas pharmacological blockade of NK-1R using antagonists such as L-733,060, L-732,138, or aprepitant suppresses tumor-cell proliferation and promotes apoptosis, thereby opposing the mitogenic effects of SP [10,16].

In addition, treatment with fosaprepitant, the prodrug of aprepitant, has been associated with increased levels of histone-bound DNA and cleaved caspase-3, consistent with the induction of apoptosis in NB cells [16]. In a nude-mouse xenograft model of NB, administration of fosaprepitant over a 14-day period also resulted in a reduction in tumor volume [16]. Interestingly, the combination therapy of fosaprepitant showed a synergistic effect with doxorubicin and etoposide in NB cells; this indicates that fosaprepitant sensitizes NB cells to chemotherapy drugs.

Moreover, the full-length isoform (*fl-TACR1*) shows a low expression profile, while truncated TACR1 splice variant (*tr-TACR1*) exhibits much higher expression levels in NB samples. NK-1R expression does not appear to be associated with specific clinical characteristics, suggesting that this receptor could represent a broadly applicable therapeutic target in neuroblastoma. Its potential relevance may extend across different disease stages and biological subtypes, regardless of the underlying tumor characteristics [42]. A molecular subtype of neuroblastoma (NB), termed C2, is associated with MYCN amplification and the concomitant expression of the transcription factors *TWIST1* and *TAC1* (*TAC1* encodes SP). Increased expression of these factors in C2 tumors results in greater SP production, driven, at least in part, by TWIST1. SP signaling via *TACR1* in tumor-associated endothelial cells promotes endothelial senescence and compromises vascular integrity. Increased *TAC1* activity has also been linked to increased tumor growth, the formation of circulating tumor cells (CTCs), and metastatic dissemination in vivo [54]. Consistent with this, patients with metastatic NB exhibit a higher frequency of *TWIST1^+^*/*TAC1^+^* CTCs in peripheral blood than patients without metastatic disease [50]. Mechanistically, *TWIST1* directly stimulates *TAC1* transcription, thereby activating the *TAC1*–*TACR1* signaling pathway and facilitating communication between neuroblastoma (NB) cells and the vascular endothelium. This interaction contributes to vascular remodeling and metastatic spread by inducing endothelial senescence and damaging endothelial intercellular junctions. Importantly, aprepitant has been shown to inhibit *TACR1*-mediated endothelial senescence, as well as tumor growth and metastatic progression in vivo, supporting the potential of NK-1R/*TACR1* blockade as a therapeutic strategy for neuroblastoma and its metastatic disease [54]. Therefore, these preclinical findings suggest the use of the drug aprepitant for the treatment of the C2 subtype of neuroblastoma, characterized by MYCN amplification and dual positivity for *TWIST1* and *TAC1* expression.

### 3.2. Pediatric Glioma

Pediatric gliomas comprise a histologically and molecularly heterogeneous group of neoplasms, classified as pediatric low-grade gliomas (pLGGs) and pediatric high-grade gliomas (pHGGs). pLGGs are the most common group of childhood brain tumors, accounting for 30–40% of new brain tumor diagnoses, with an estimated incidence of 2–3 per 100,000 in Western populations. Abnormal activation of the RAS/MAPK pathway is a nearly universal feature, to the extent that these tumors have been described as a “single-pathway disease” [55]. Surgery remains the mainstay of treatment. When resection is not feasible, chemotherapy has been the standard second-line treatment, with 5-year PFS rates around 50%. The advent of targeted therapies against the RAS/MAPK pathway has changed the therapeutic landscape, with results superior to first-line chemotherapy in pLGG with BRAF V600E [55]. pHGGs such as diffuse midline glioma, H3 K27-altered, have a 2-year survival below 10% and are inoperable. Unlike pLGGs, pHGGs are immunologically “cold” tumors, which limits conventional immunotherapy options, except in the hypermutated subgroup due to biallelic mismatch repair deficiency [56]. pLGGs exhibit a complex TME containing approximately 50% non-tumor cells, such as immune cells and neovessels [57], with VEGF levels elevated. Glioma cells, via the MAPK-NF-kB axis, produce various cytokines such as CCL2 and CCL4, which are responsible for recruiting microglia or circulating monocytes (with CCR2 or CCR5 receptors) that promote inflammation, which serves the oncogenic process [55,58]. Indeed, murine models fail to develop glioma in the absence of immune cells [57]. Among all pLGGs, those with KIAA1549: BRAF exhibit a more inflammatory microenvironment, in contrast to those with BRAFV600Em [55,56]. Pediatric HGGs—particularly DMG—show a lymphocytic infiltration nearly identical to that of healthy tissue (<3% CD45^+^ leukocytes), robust expression of TGF-β, low expression of PD-L1 and NKG2DL, and a pattern of tumor-associated macrophages lacking marked M1/M2 polarization [56]. Oncogene-induced senescence (OIS) is a well-known phenomenon in pLGG and pilocytic astrocytoma with its classic driver fusion KIAA1549: BRAF; markers of senescence are detected, such as acid β-galactosidase expression, cell cycle arrest in the G1 phase, heterochromatin foci associated with senescence, and activation of tumor suppression mechanisms (p53/p21 and p16INK4a/RB). In fact, p16INK4a dysfunction, present in 6–20% of pLGG, is a sign of malignant acceleration and exit from senescence [55,57]. TIS is a phenomenon with implications for the treatment of pLGG; treatment-related stress enhances SASP, which acts as a tumor “fertilizer”: pro-angiogenic vascular endothelial growth factor (VEGF), pro-invasive metalloproteinases (MMPs), immunosuppressive (via granulocyte-macrophage colony-stimulating factor (GM-CSF) and recruitment of circulating mesenchymal cells), and mitogenic (IL-6, required for glioma formation from glial stem cells) [59].

It is classically known that the glioblastoma (GB) samples studied (10 out of 10) express NK-1R and, in the case of the astrocytoma samples, in 9 out of 12. In addition, the expression of NK-1R was less marked or extensive in astrocytoma than in GB [45]. In addition, glioma cell lines (e.g., LN71, LN229, LN319, LN405, U-373 MG, U-251 MG, DBTRG-05 MG, UC-11 MG, SNB-19, GAMG, U-87 MG) express NK-1R [60]. The number of NK-1R is lower in glioma cell line cultures than in primary gliomas, and a worse prognosis and advanced tumor stages were related to the most malignant phenotypes (NK-1R overexpression) [45,61]. Glioma cells have been reported to express between 40,000 and 60,000 NK-1R per cell in UC-11 MG glioma [62]. In GB samples, the number of NK-1R is higher in the tumors than in the surrounding normal tissues, and NK-1R was found in blood vessels located within the tumor [45].

Studies in GAMG and U-87 MG glioma cells have shown that SP, together with the NK-1R-FL and NK-1R-T isoforms, can be detected in both the nucleus and cytoplasm. The NK-1R-FL isoform is predominantly nuclear, while NK-1R-T is more abundant in the cytoplasmic compartment [39]. Functional studies further indicate that TACR1 (NK-1R) expression is required for the survival of GAMG and U-87 MG glioma cells, whereas this gene appears to be dispensable for the viability of normal, non-malignant cells. Conversely, the *TAC1* gene is not essential for the survival of these glioma cell lines [39]. In glioma cells, activation of NK-1R by SP promotes proliferative signaling and mitogenic activity [10,63,64]. In U-373 MG cells, SP stimulation of the NK-1R rapidly increases inositol triphosphate (IP3) production and elevates intracellular Ca^2+^ levels. This signaling cascade also induces early expression of the c-Fos and c-Jun genes, resulting in activation of the transcription factor AP-1 (activator protein-1) and increased DNA synthesis [63]. Furthermore, SP upregulates c-Myc mRNA and protein levels, which are important for the transition of glioma cells from the G1 to the S phase of the cell cycle. Activation of NK-1R by SP also triggers the mitogen-activated protein kinase ERK1/2 (MAPK) pathway, further supporting the proliferative response of glioma cells [65]. In U-373 MG glioma cells, SP induced tyrosine phosphorylation of several proteins, including EGFR (an essential regulator of SP/NK-1R), which activates the EGFR complex containing the adaptor proteins Sos, Shc, and Grb2, but not c-Src, thereby increasing ERK2 kinase activity [66].

SP, at nanomolar concentrations, increases the proliferation of glioma cells while NK-1R antagonists (L-733,060, L-732,138, CP-96,345 and aprepitant) counteract SP-mediated glioma cell proliferation in a concentration-(micromolar) and time-dependent manner [60]. The antiproliferative effects of NK-1R antagonists appear to be associated with their receptor-binding properties. In GAMG glioma cells, L-733,060 showed the greatest inhibitory potency, followed by aprepitant and L-732,138 [60]. Supporting the antitumor potential of NK-1R blockade, the antagonists MEN-11,467 and MEN-11,149 have been reported to suppress tumor growth in human glioma xenograft models [67]. Mechanistically, SP promotes survival signaling in glioma cells by inducing Akt phosphorylation, thereby contributing to the inhibition of apoptosis [68]. Conversely, NK-1R inhibition with aprepitant triggers apoptotic cell death in GAMG glioma cells [19]. Similarly, L-733,060 reduces the constitutive activity of NK-1R-dependent Akt kinase and potentiates apoptosis, accompanied by caspase-3 activation and proteolytic degradation of poly (ADP-ribose) polymerase (PARP). The signaling mechanism underlying NK-1R-mediated Akt activation involves Src and phosphoinositide 3-kinase (PI3K), and epidermal growth factor receptor (EGFR) signaling also contributes to this pathway [68]. Furthermore, basal Akt activity has been associated with a poor prognosis in glioma [68]. Moreover, it has been reported that the combination of temozolomide plus aprepitant increase cytotoxicity in the GAMG glioma cells [69]. Moreover, in human glioma U87-MG, SP increases glioma cells proliferation in a dose dependent manner (5–500 nM). In contrast, aprepitant (Table 1) reduced glioma cell proliferation, migration, and invasion in a dose-dependent manner (IC_25_: 38.5 μM, IC_50_: 77 μM) and decreases the expression of pro-tumor mediators such as VEGF, NF-kB, TNF-α, IL-6, IL-8, CCL3, CXCL3 and TRIM5, indicating a suppression of the pathways of angiogenesis, inflammation and chemotaxis [46]. It should be noted that aprepitant is a small lipophilic molecule that crosses the blood–brain barrier. In fact, its clinical use is for CINV, acting on the NK-1R in the vomiting centers within the CNS to block its activation by SP released as an unwanted consequence of chemotherapy [7]. Thus, aprepitant may be useful in treatments for brain tumors such as pediatric glioma [60].

### 3.3. Craniopharyngioma

Craniopharyngioma is a tumor that originates in the craniopharyngeal duct; although rare in the general population, it accounts for 10% of all childhood brain tumors. Although it is a benign tumor, its close location to the optic tract and hypothalamus is associated with significant morbidity [70]. Surgery and radiation therapy (RT) are the standard treatments for this condition; unfortunately, despite these treatments, the recurrence rate is high, and these procedures also cause damage to the hypothalamus [71]. In addition, irreversible sequela persists, such as hypothalamic obesity and diabetes insipidus, among other factors that impair patients’ quality of life [72]. The oncogenic mechanism underlying Adamantinomatous craniopharyngioma (ACP) is based on a senescence-associated secretory phenotype, which is linked to the production of paracrine biomarkers that create an inflammatory and angiogenic microenvironment that promotes ACP progression [73]. ACP is the most common type in children. It consists of cellular clusters of quiescent tumor cells that carry a β-catenin mutation and exhibit marked SASP characteristics [74]. The characteristic oncogenic signaling pathway of ACP is the WNT pathway, which, through the production of midkine (MDK) promotes an immunosuppressive TME in this type of cancer [75]. The ACP microenvironment contains inflammatory cysts, tumor macrophages, glial reaction, and neovessels, with the detection of abundant markers such as interleukins, MMP, fibroblast growth factor (FGF), and bone morphogenic protein (BMP). This paracrine activity supports the invasive nature of ACP toward hypothalamic regions, which may contribute to the limited efficacy of surgery and radiotherapy in controlling hypothalamic invasion.

Furthermore, it is well known that SP and NK-1R are involved in inflammation and cancer promotion and progression [9]. The SP/NK-1R signaling system is known to be markedly overexpressed in ACP compared to normal pituitary tissue [47]. SP is widely distributed in ACP lesions, with a predominant nuclear localization in tumor cells. Nuclear expression of SP is also observed in areas of glial reactivity and in endothelial cells. In contrast, NK-1R expression is primarily associated with the inflammatory and stromal components of the TME, particularly within areas of glial reaction, where it is detected predominantly in the nuclei and cell membranes of inflammatory cells and, to a lesser extent, in the cytoplasm. NK-1R is also present in endothelial cells and basement membrane-associated fibroblasts within newly formed tumor vessels, while neoplastic cells show little or no significant NK-1R expression. Taken together, these findings suggest that the SP/NK-1R axis is particularly active within the inflammatory and angiogenic compartments of the ACP microenvironment, including glial cells, endothelial cells, and perivascular fibroblasts [47].

These findings suggest that SP and the NK-1R play a role in the pituitary gland and in ACP. In contrast, it is known that NK-1R antagonists counteract the pathophysiological effects of SP and open the door for future clinical trials of treatment with NK-1R antagonists, such as the drug aprepitant, in patients with ACP.

### 3.4. Hepatoblastoma

Hepatoblastoma (HB) is the most common primary malignant liver tumor in children, accounting for approximately 80% of liver tumors in this age group. It is considered an embryonic tumor that arises from hepatoblasts because of disruptions in normal liver development. This arrest at the immature stage promotes the transition to a secretory phenotype, with the production of cytokines, midkine (MDK), or FGF [75,76]. HB’s overall incidence is approximately 1.5 cases per million children per year. Overall survival exceeds 80–90% in localized disease but drops to 50–70% in metastatic and high-risk disease. Treatment includes polychemotherapy regimens and aggressive surgeries that may include liver transplantation; this, combined with a very early median age at presentation (18 months), leads to significant toxicity [77]. HB is characterized by an immunologically “cold” TME resulting from a high infiltration of MARCO low macrophages, which exert an immunosuppressive effect via CD44, PDGF, TNFR, and PLXNB2. “Erythroblastic islands” composed of VCAM1^+^ macrophages and erythroid cells that suppress immunity via the LGALS9/TIM3 axis on dendritic cells have also been described, inversely correlating with survival [75]. The oncogenic signaling pathway characteristic of HB and other tumors such as ACP is the WNT pathway, which, through the production of MDK, promotes an immunosuppressive TME in these types of cancers [75]. HB expresses angiogenic mediators such as hypoxia-inducible factor (HIF) and VEGF, and develops new blood vessels that may limit delivery of chemotherapy drugs and facilitate the arrival of mesenchymal and immunomodulatory cells [76].

SP/NK-1R in HB has been reported to show that both tumor samples and established HB cell lines, including HepT1, HepG2, and HuH6, express full and truncated forms of NK-1R (Table 1). Notably, NK-1R-T is expressed at higher levels in HB cells than in normal, non-tumor cells [14]. Nanomolar concentrations of SP promote HB cell proliferation, while pharmacological inhibition of NK-1R with L-733,060, L-732,138, or aprepitant suppresses this proliferative response [14]. At micromolar concentrations, these antagonists induce apoptotic cell death in HB cells and, consequently, prevent the mitogenic effects triggered by SP. This pro-apoptotic response is accompanied by the cleavage of poly (ADP-ribose) polymerase (PARP) and caspase-3, providing evidence of activation of the apoptotic pathway [14] (Table 1).

In an HB xenograft model in nude mice, daily administration of aprepitant at 80 mg/kg for 24 days resulted in a significant reduction in both tumor volume and mass. This treatment was also associated with fewer Ki-67-positive cells and lower tumor vascular density, without any notable adverse effects on the animals’ overall condition or body weight throughout the treatment period [14]. These findings support the potential of aprepitant as a therapeutic approach for HB. Mechanistic studies in the human HB cell lines HepT1, HepG2, and HuH6 further indicate that aprepitant interferes with Akt- and Wnt-dependent signaling. The treatment reduced p70S6K and 4E-BP1/2 phosphorylation and suppressed canonical Wnt signaling, ultimately limiting HB cell proliferation. The magnitude of this response was associated with elevated baseline Wnt activity, either as a result of β-catenin mutations or extracellular Wnt stimulation. Furthermore, inhibition of Wnt signaling was potentiated by disruption of the FOXM1–β-catenin interaction. Aprepitant also decreased the expression of several markers associated with hepatic pluripotency, including AFP, CD13, SOX2, NANOG, and OCT4, and also reduced sphere-formation ability (SFA) under cancer stem cell culture conditions [78]. Thus, the NK-1R antagonist aprepitant could be used for the treatment of HB.

### 3.5. Retinoblastoma

Retinoblastoma (RB) is an embryonic tumor that originates from the cone precursors of the retina. Its incidence is 1 case per 16,000 births, and bilateral cases are a leading cause of blindness in children [79]. It arises from biallelic inactivation of the RB1 tumor suppressor gene, which disrupts cell cycle regulation and leads to uncontrolled cell proliferation [79,80,81]. RNA-Seq analysis has identified cellular heterogeneity in RB with minimal lymphocytic infiltration, populations of tumor-associated macrophage type 2 (TAM2), which promote immunotolerance and are enriched with MMP-9, and cancer-associated fibroblasts (CAFs) that facilitate the spread of the neoplasm within the vitreous. A high abundance of pro-tumor markers such as IGFBP-5, MDK and angiopoietin-like proteins (ANGPTL) was also detected [80,82].

Expression of NK-1R has been detected in both retinoblastoma tumor specimens and established retinoblastoma cell lines, including WERI-Rb-1 and Y-79 [12] (Table 1). Experimental studies have demonstrated that SP promotes the growth of retinoblastoma cells, whereas pharmacological blockade of NK-1R with L-733,060, L-732,138, or aprepitant markedly reduces this proliferative response. NK-1R inhibition is also associated with apoptotic cell death and prevents the mitogenic effects induced by SP [11,12,19] (Table 1). Collectively, these findings support further investigation of aprepitant and other NK-1R antagonists as potential therapeutic options for retinoblastoma.

### 3.6. Osteosarcoma

Osteosarcoma (OS) is the most common primary malignant bone tumor, characterized by malignant cells that produce osteoid or immature bone tissue, and typically arises from the malignant transformation of cells of mesenchymal lineage at an undefined stage of differentiation toward osteoblasts. Molecularly, they are characterized by a loss of the tumor suppressor genes CDKN2A, PTEN, TP53, and RB1. Its incidence is 3–4.5 cases per million people per year. It primarily affects the long bones of the lower limbs in adolescents and young adults. Amputation of the limb is a common treatment, and survivors often experience orthopedic complications [83]. With polychemotherapy and surgery, event-free survival rates of 60–70% are achieved in localized disease [83]. However, outcomes have remained virtually stagnant since the 1980s, with 5-year survival rates below 30% in metastatic disease and below 20% in relapsed disease [83].

OS shows a complex TME [83,84]. The predominant infiltrating cells are TAM, with a clear predominance of the M2 phenotype, which promote metastasis and angiogenesis via IL-10 or TGF-β. OS also shows low CD8^+^ lymphocyte infiltration, with high expression of PD-1 and TIM-3, indicating exhaustion. A high proportion of regulatory T cells (Tregs) are present; these secrete IL-10, IL-35, and TGF-β, suppress the effector response, and are associated with a poorer prognosis. Myeloid-derived suppressor cells (MDSCs) are also found in the OS TME. Recruited via the SDF-1/CXCR4 axis, they suppress T-cell function through arginase-1, iNOS, and ROS, and promote the pulmonary premetastatic niche. The dysfunction of the natural killer (NK) and dendritic cells present in the tumor is evident. In OS, senescent cells in the TME, through the SASP, release proinflammatory cytokines (IL-6, IL-8, TNF-α) and MMPs that paradoxically promote the proliferation of residual tumor cells, facilitate EMT and promote angiogenesis [85].

The SP/NK-1R axis has also been investigated in osteosarcoma (OS). MG-63 cells have been shown to express NK-1R, with substantially higher *TACR1* expression in these cells than in HEK293 cells or normal human fibroblasts. In fact, HEK293 cells showed approximately half the *TACR1* expression detected in MG-63 cells, while fibroblasts exhibited only minimal NK-1R expression. At nanomolar concentrations, SP stimulates the proliferation of MG-63 OS cells [15] (Table 1). Conversely, pharmacological inhibition of NK-1R by L-733,060, L-732,138, or aprepitant suppresses SP-induced proliferation and promotes apoptotic cell death in MG-63 cells [15]. The IC_50_ values for the antitumor effects of L-733,060, L-732,138, and aprepitant were 14.55, 58.61, and 28.65 μM, respectively. Interestingly, combining L-733,060 with conventional chemotherapeutic agents, such as doxorubicin, ifosfamide, or cisplatin, potentiated their antitumor effects. In addition, pretreatment of HEK293 cells with L-733,060 partially protected them from cisplatin- and ifosfamide-induced cytotoxicity [15].

Furthermore, in a nude mouse xenograft model of osteosarcoma, subcutaneous administration of fosaprepitant at 80 mg/kg/day for 28 days significantly reduced tumor volume compared with untreated controls [15]. Therefore, these findings support the further investigation of aprepitant as a candidate for repurposing in OS.

### 3.7. Rhabdoid Tumor

Rhabdoid tumors (RTs) are rare, highly malignant embryonal neoplasms that occur predominantly in infants and young children. Despite intensive multimodal treatment, outcomes remain unsatisfactory, while conventional therapeutic approaches are frequently associated with substantial toxicity and significant late complications. Based on their anatomical location, these tumors include atypical teratoid/rhabdoid (AT/RT) tumors of the central nervous system, renal rhabdoid tumors (RRTs), and extrarenal or malignant rhabdoid tumors (RMTs) originating in the liver and soft tissues [86]. At the molecular level, RTs are primarily defined by the biallelic inactivation of SMARCB1 or SMARCA4, genes that encode components of the SWI/SNF chromatin remodeling complex. The loss of these proteins results in profound epigenetic alterations, including increased dependence on H3K27 trimethylation. Molecular profiling has further classified AT/RT tumors into three main subgroups: AT/RT-TYR, AT/RT-SHH, and AT/RT-MYC, with the MYC subgroup exhibiting distinctive immunological characteristics. These tumors may contain dysfunctional CD8^+^ memory effector T cells characterized by the expression of immune checkpoint receptors such as PD-1, TIM-3, and LAG-3 [87]. Limited knowledge of the molecular landscape and TME of AT/RT tumors has hampered the development of effective therapies. Consequently, the considerable toxicity and long-term complications associated with current chemotherapy, coupled with the lack of consistently effective standard treatments, underscore the urgent need to explore novel, molecularly targeted strategies capable of improving survival and reducing treatment-related morbidity [41].

RT cell lines G-401, BT-12, and CHLA-266 express both *TACR1* and *TAC1*. Across all three models, the *tr-TACR1* isoform was considerably more abundant than *fl-TACR1*, and *TACR1* expression showed no clear relationship with clinical features. RT-PCR analysis of six rhabdoid tumor specimens confirmed the presence of *TACR1* transcripts and reproduced the expression pattern observed in AT/RT and renal rhabdoid tumor (RTK) cell lines, with *tr-TACR1* predominating over *fl-TACR1*. Among the tumor samples, *tr-TACR1* levels were highest in the hepatic rhabdoid tumor and lowest in the AT/RT specimens, whereas *fl-TACR1* expression remained minimal across all samples examined. Interestingly, TAC1 expression displayed a markedly different pattern: the renal rhabdoid tumor showed the highest *TAC1* levels, while little to no *TAC1* expression was detected in the remaining tumor tissues [41].

The antitumor activity of aprepitant was evaluated in the rhabdoid tumor cell lines G-401, BT-12, and CHLA-266. HepG2 hepatoblastoma cells were included as a positive control, whereas primary dermal fibroblasts from neonatal and adult donors served as negative controls. Cells were incubated with progressively higher concentrations of aprepitant (10–100 μM) for 48 h. All three RT models exhibited a concentration-dependent reduction in proliferation following treatment, whereas neither neonatal nor adult fibroblasts showed a comparable response (Table 1). The inhibitory effect observed in G-401, BT-12, and CHLA-266 cells was similar to that detected in HepG2 cells [41]. The potential benefit of combining aprepitant with cisplatin was subsequently investigated in these tumor models. A statistically significant additive antiproliferative effect was detected in HepG2 and CHLA-266 cells, while comparable trends were observed in G-401 and BT-12 cells but did not reach statistical significance [41]. SP (200 nM, 48 h) significantly attenuated the antiproliferative effect of aprepitant in BT-12 cells; a similar, non-significant trend was observed in G-401 and CHLA-266 [41]. SP significantly inhibited the anti-proliferative effect of aprepitant in BT-12. Similar effects were observed in G-401 and CHLA-266, but the difference was not statistically significant. Following treatment with aprepitant, an increase in the proportion of cells in the early and late stages of apoptosis was observed, as well as strong expression of PARP-1 cleavage products in rhabdoid tumor cells. Thus, aprepitant induces apoptosis in RT cells [41].

### 3.8. Acute Lymphoblastic Leukemia

Childhood acute lymphoblastic leukemia (ALL) is the most common type of cancer in childhood. In the United States, leukemias account for approximately one-third of all malignancies diagnosed in children under 15 years of age, and ALL constitutes the majority of pediatric leukemia cases. Advances in treatment have substantially improved outcomes over time. Current estimates indicate that children with ALL can achieve a 10-year disease-free survival rate of approximately 80% and a 10-year overall survival rate approaching 90% [88]. ALL is the most common childhood cancer, with more than 150,000 new cases worldwide each year. It is characterized by the uncontrolled proliferation of lymphoid progenitor cells (lymphoblasts), which displace normal hematopoiesis in the bone marrow and infiltrate healthy tissues. Treatment based on polychemotherapy, optimization of hematopoietic transplantation techniques, and supportive care has achieved high cure rates; however, cases with a poor biological prognosis (BCR: ABL1, KMT2A rearrangements, E2A-HLF) continue to exist, requiring the use of bispecific antibodies or CAR-T therapy [89]. ALL cannot be understood solely in terms of intrinsic (genetic/epigenetic) alterations in the tumor cell. This is known as leukemia-induced stromal senescence: leukemic blasts block the differentiation of mesenchymal cells into osteoblasts and, via RANKL, induce osteoclastic dominance, thereby facilitating invasive bone marrow lesions. Furthermore, leukemia cells produce TGF-β, IL-6, TNF-α, IL-1β and VEGF, and activate Wnt and Notch signaling under HIF-dependent hypoxic conditions, which prevent the differentiation of hematopoietic stem cells and recruit adipocytes and neovessels that supply the leukemic microenvironment with energy and nutrients [90,91].

The SP/NK-1R signaling axis has also been investigated in ALL. Normal human blood T lymphocytes have been reported to carry approximately 7000–10,000 NK-1R molecules per cell, whereas human IM-9 B-lymphoblasts express substantially higher levels, ranging from 25,000 to 30,000 receptors per cell [92]. Notably, NK-1R mRNA expression in ALL cells is approximately 30-fold greater than that detected in normal cells. In addition, SP has been identified in human leukemic blast cells from patients with ALL (Table 1). Together, these findings support a potential role for the SP/NK-1R axis in autocrine and/or paracrine signaling mechanisms in ALL [93]. Moreover, in patients with ALL, SP expression was demonstrated in 41.2%. In pre-B ALL, SP expression was observed in 25.0%. In patients with T-ALL, SP expression was demonstrated in 81.2%. The percentage of immunopositive cells in SP-positive cases ranged from 79.8% to 97.3%.

Evidence suggests that SP expression at diagnosis may be associated with a higher risk of treatment failure in children with ALL, as the presence of SP-positive leukemic blasts has been linked to subsequent disease relapse [94]. Given that SP is involved in promoting malignant cell growth, its expression may have potential value as a prognostic or risk-associated biomarker in ALL. At the cellular level, T-ALL BE-13 and B-ALL SD-1 cell lines express NK-1R isoforms and respond to SP with increased proliferation. Conversely, pharmacological blockade of NK-1R with L-733,060, L-732,138, or aprepitant suppresses this proliferative response and promotes apoptosis [13] (Table 1). In pre-B ALL Nalm-6 cells, aprepitant has been shown to induce apoptosis by inhibiting the PI3K/Akt signaling pathway [95]. This treatment also increases the levels of the cyclin-dependent kinase inhibitors p27 and p21, leading to cell cycle arrest in the G1 phase [96]. Furthermore, genetic silencing of NK-1R in BE-13 and SD-1 cells reduces cell viability, which is consistent with the involvement of apoptotic mechanisms in the response to NK-1R depletion [13].

Thus, it is possible to obtain the same therapeutic effect (the death of T-ALL BE-13 and B-ALL SD-1 cells by apoptosis) by two different strategies: pharmacological (using NK-1R antagonists: L-733,060, L-732,138, aprepitant) or genetic (applying the siRNA *TACR1* method) [13]. Furthermore, the prognosis for relapsed/refractory acute lymphoblastic leukemia (R/R ALL) remains unfavorable due to chemoresistance, making the search for new therapeutic strategies urgent and necessary.

Overexpression of NK-1R in ALL has generated increasing interest in this receptor as a potential therapeutic target, especially in patients with refractory disease. Aprepitant has demonstrated marked antiproliferative and antileukemic effects in both primary ALL samples and doxorubicin-resistant cell models, suggesting that NK-1R inhibition could help overcome resistance to conventional chemotherapy [97]. Interestingly, its mechanism of action in ALL appears to differ from the apoptotic responses previously described in other malignancies. In this context, aprepitant activates a form of mitochondria-driven necroptotic cell death [98]. The process involves the release of calcium from the endoplasmic reticulum (ER) and the induction of ER stress, followed by mitochondrial calcium accumulation, increased production of reactive oxygen species (ROS), and mitochondrial fragmentation. These events ultimately promote the phosphorylation and activation of key proteins associated with necroptosis, such as RIP1K, RIPK3, and MLKL [97]. Furthermore, the antileukemic activity of aprepitant has been confirmed in vivo using a xenograft mouse model of ALL, providing further support for its potential therapeutic application [97].

### 3.9. Acute Myeloid Leukemia

Acute myeloid leukemia (AML) is characterized by the uncontrolled accumulation of malignant myeloid cells in the bone marrow. Despite advances in cancer treatment, long-term disease-free survival among patients with AML remains unsatisfactory, with only limited progress in survival outcomes achieved in recent decades. AML is a neoplasm characterized by the clonal proliferation of myeloid progenitor cells, which displace normal hematopoiesis in the bone marrow and peripheral blood. In children, the incidence is approximately 7 per million. The 5-year survival rate is 70% [99]. As with its lymphoid counterpart, AML interacts with mesenchymal cells and the endosteal niche via VEGF/VEGFR or CXCL12/CXCR4, among others, to create a microenvironment favorable to leukemogenesis. In AML, disruption of neurogenic regulation via the beta3-adrenergic system—which normally regulates myeloid proliferation—has been observed [100,101]. The immunopermissive TME is also characteristic of AML, with a significant presence of Tregs and TAM2 cells, alongside the presence of the inhibitory molecules IL-1, IL-6, IL-10, TGF-β and IDO [102].

It is known that KG-1 and HL-60 AML cell lines express two isoforms of NK-1R: full length and truncated (Table 1). The SP/NK-1R axis has been implicated in the biology of AML. The KG-1 and HL-60 AML cell lines exhibit higher expression of NK-1R-T than lymphocytes obtained from healthy donors. Exposure to nanomolar concentrations of SP promotes proliferation in both AML models, whereas pharmacological inhibition of NK-1R with L-733,060, L-732,138, CP 96-345, or aprepitant suppresses leukemic cell growth [17] (Table 1). In the acute promyelocytic leukemia cell line NB4, aprepitant induces upregulation of p21 and p73 while reducing c-MYC and hTERT expression, and additionally enhances the cytotoxic activity of vincristine and arsenic trioxide [48]. The antileukemic effects of NK-1R antagonists appear to depend on receptor expression in AML cells and are associated with apoptotic cell death involving caspase-3 activation [17,48] (Table 1). Consistent with these in vitro findings, daily intraperitoneal administration of high-dose fosaprepitant to NOD scid gamma mice bearing HL-60 xenografts increased median survival from 4 days in untreated controls to 7 days [17] (Table 1). Analysis of three AML cell lines revealed moderate-to-strong immunoreactivity for both SP and NK-1R, whereas neither marker was detected in samples obtained from 10 healthy volunteers. Two NK-1R receptor variants have been identified: NK-1R-F, of approximately 46 kDa, and NK-1R-T, of approximately 38 kDa. In K562 cells, pharmacological inhibition of NK-1R with SR140333 promotes a pro-apoptotic response characterized by increased levels of Bax and Bim, activation of caspases-3 and -9, and PARP cleavage, together with a reduction in the anti-apoptotic proteins Bcl-2 and Bcl-xL [98]. Mechanistically, NK-1R blockade promotes mitochondrial dysfunction through increased production of reactive oxygen species (ROS). This response appears to involve a rapid transfer of Ca^2+^ from the endoplasmic reticulum to mitochondria, resulting in mitochondrial calcium overload, oxidative stress, and subsequent apoptotic cell death. Notably, both SR140333 and aprepitant induced more pronounced and sustained cytoplasmic and mitochondrial Ca^2+^ fluxes than hydrogen peroxide (H_2_O_2_). Beyond its direct antileukemic effects, NK-1R inhibition has also demonstrated analgesic potential in models of myeloid leukemia-associated bone pain, apparently through simultaneous reduction in inflammation and induction of apoptosis [98].

## 4. NK-1R Is Essential for Viability of Cancer Cells

The functional relevance of the SP/NK-1R axis in GAMG and U-87 MG glioma cells was investigated using siRNA-mediated gene silencing of *TACR1*, which encodes NK-1R, and *TAC1,* which encodes SP. Suppression of *TACR1* markedly reduced glioma cell proliferation, whereas silencing *TAC1* had no comparable effect. The loss of viable glioma cells following *TACR1* knockdown was associated with both apoptotic and necrotic cell death. Interestingly, depletion of *TACR1* in primary human fibroblasts did not significantly affect their viability. These findings indicate that NK-1R expression is important for the survival and proliferation of GAMG and U-87 MG glioma cells, while normal human fibroblasts appear to be considerably less dependent on this signaling pathway [39].

NK-1R-specific target: Since healthy cells do not rely heavily on the NK-1R receptor, blocking this receptor effectively eliminates cancer cells without causing the severe and widespread toxicity that is usually associated with traditional chemotherapy. In line with this, it has been reported that, using the *TACR1* gene silencing method with siRNA in the T-ALL BE-13 and B-ALL SD-1 cell lines, transfected cells significantly decreased their proliferation compared to non-transfected cells, and that the transfected cells died by apoptosis [13]. Similar findings have been reported in human cell lines of small cell lung carcinoma H-69 and non-small cell lung carcinoma COR-L23 [103], MEL HO and COLO 679 melanoma cell lines [104], and in BT-474, MCF-7 and MDA-MB-468 breast cancer cell lines [105]. These findings indicate that *TACR1* silencing reduces viability in the cancer cell lines studied to date.

## 5. The SP/NK-1R System Is Involved in Cancer Promotion and Progression

It has been reported that SP is an universal mitogen in cancer cells [9,38] and, in a concentration-dependent manner at nanomolar concentration, induces tumor cell proliferation by activating mitogen-activated protein kinases (MAPKs: p38 MAPK, ERK1/2, JNKs), NF-κB, activator protein-1 (AP-1), composed of c-Fos and c-Jun, and c-Myc, and also promotes anti-apoptotic activity in tumor cells through the phosphatidylinositol 3-kinase (PI3K)/protein kinase B (PKB/Akt)/mTOR signaling pathway [9,60]. In tumor cells, SP promotes the Warburg effect by promoting the breakdown of glycogen to glucose-6-phosphate, which fuels glycolysis in tumor cells [9,60] and favors blebbing and the release of MMP-2 and MMP-9, which hydrolyze the extracellular matrix (ECM), facilitating the invasion and migration of tumor cells and leading to metastasis [9,60]. Thus, the importance of the NK-1R in cancer cells is due to the fact that it regulates multiple signaling pathways involved with the promotion and progression of cancer. It is important to note that, as with genetic silencing of *TACR1*, it is possible to pharmacologically block NK-1R using NK-1R antagonists, such as aprepitant, in a concentration-dependent manner to achieve antitumor activity [9].

## 6. The SP/NK-1R System Is Involved in Neurogenic Inflammation and Angiogenesis

The relationship between inflammation and cancer has been known for over a century, dating back to Rudolf Virchow’s 1863 observation of the presence of leukocytes in neoplastic tissues. Subsequent research has supported the idea that the inflammatory environment associated with tumors can promote malignant progression rather than generate an effective antitumor response. In particular, tumor-associated inflammatory cells and the cytokines they produce can facilitate tumor growth, disease progression, and immunosuppression [106]. SP is considered a key mediator of neurogenic inflammation [8]. This neuropeptide is released primarily from the nerve endings of primary sensory neurons and is involved in inflammation processes, with nerve fibers containing SP frequently located near blood vessels.

SP is primarily released from the peripheral nerve endings of primary sensory neurons and plays a significant role in the development of neurogenic inflammation. Nerve fibers containing SP are frequently located near blood vessels, facilitating the release of this neuropeptide into peripheral tissues. Inflammatory cells, including macrophages, eosinophils, lymphocytes, and dendritic cells, can also produce SP and express its receptor, NK-1R. Furthermore, endothelial cells express NK-1R, enabling them to respond directly to SP. Activation of NK-1R by SP contributes to several features of the inflammatory response, such as vasodilation, increased vascular permeability, plasma leakage, and the recruitment of leukocytes to sites of tissue injury. SP can also promote leukocyte migration and accumulation, stimulate mast cell degranulation, and modulate the activity and function of immune cells [8]. Furthermore, SP, after binding to NK-1R, which is overexpressed in inflammatory cells in the presence of neuroinflammation, regulates neurogenic inflammation mediated by the release of IL-1β, IL-6, IL-8, TNF-α and GM-CSF [8,107,108]. Activation of neutrophils by SP leads to the formation of reactive oxygen and nitrogen species, including superoxide anion, hydrogen peroxide and nitric oxide through different signaling pathways [109]. In addition, SP mediates the induction of AP-1 in A549 human lung adenocarcinoma cells through ROS, which could contribute to the expression of pro-inflammatory cytokines [110].

The Janus kinase/signal transducer and activator of transcription (JAK/STAT) cascade is a key intracellular signaling network that regulates a wide range of essential biological functions. By transmitting signals from cell surface receptors to the nucleus, this pathway rapidly controls the transcription of genes involved in processes such as inflammation, cell survival, proliferation, and tumor development. Accumulating evidence indicates that activation or aberrant regulation of JAK/STAT signaling contributes to the pathogenesis and progression of various malignancies and autoimmune disorders [111].

SP has been shown to activate the JAK/STAT signaling cascade in human colonocytes expressing NK-1R. In NCM460-NK-1R cells, SP stimulation induces concentration- and time-dependent phosphorylation of JAK2, STAT3, and STAT5, subsequently promoting COX-2 expression and prostaglandin E2 (PGE2) synthesis. Consistent with these in vitro findings, experimental colitis in mice is associated with increased COX-2 expression in colonic tissue, an effect that can be reversed by treatment with the NK-1R antagonist CJ-12.255. Taken together, these observations indicate that SP regulates COX-2 and PGE2 production in human colonocytes through activation of the JAK2–STAT3/5 signaling pathway [112].

Angiogenesis is a multi-step biological process that begins with the activation and proliferation of endothelial cells and culminates in the formation and maturation of new blood vessels. In inflammatory tissues, this process is accompanied by the progressive development of neurovascular regulatory mechanisms [113]. Angiogenesis is a key feature of tumor progression and is frequently accompanied by increased tissue innervation and NK-1R expression [113]. The NK-1 receptor is also present in blood vessels located both within and around tumor lesions. By activating the vascular NK-1 receptor, SP can modulate the structure and function of the tumor vasculature, increasing local blood flow and contributing to stromal development. These vascular effects can create a microenvironment that favors tumor progression and facilitates metastatic spread [45].

SP induces marked neovascularization in vivo, and the addition of SP to cultured endothelial cells, including human umbilical vein cells (HUVECs), increased the proliferation of both cell lines in a concentration-dependent manner. A selective NK-1R agonist induced a similar proliferative effect, while selective NK-2R and NK-3R agonists had no effect. Conversely, two NK-1R antagonists blocked the response to SP. Therefore, SP may directly stimulate the neovascularization process, likely by inducing endothelial cell proliferation. Thus, SP could play a key role in the trophic effect produced by the activation of the efferent function of peripheral terminals of primary sensory neurons [32]. In addition, during neurogenic inflammation, SP is released from sensory nerves, which, by binding to the NK-1R receptor of endothelial cells, increases angiogenesis. In contrast, NK-1R antagonist SR140333 inhibits angiogenesis [114].

On the other hand, hemokinin-1 (HK-1) has also been identified as an endogenous mediator capable of promoting angiogenesis through NK-1R activation. In human umbilical vein endothelial cells (HUVECs), HK-1 induces concentration-dependent proliferation, migration, adhesion, and capillary tube formation, while similar proangiogenic effects have been demonstrated in vivo. These responses are markedly reduced by selective NK-1R antagonists, supporting the involvement of this receptor in HK-1-mediated angiogenic signaling [115]. At the molecular level, HK-1 promotes ERK1/2 phosphorylation and stimulates nitric oxide production, along with increased expression of endothelial nitric oxide synthase (eNOS) and vascular endothelial growth factor (VEGF) in HUVECs [115]. VEGF is a major regulator of vascular development that promotes endothelial cell proliferation and survival, increases vascular permeability, and facilitates endothelial migration [116]. Consequently, elevated VEGF expression within tumors may contribute to increased angiogenesis, tumor growth, and metastatic progression [117].

## 7. The SP/NK-1R System as a Mediator of Onconeuroimmune Crosstalk in the TME

**Figure 1:**
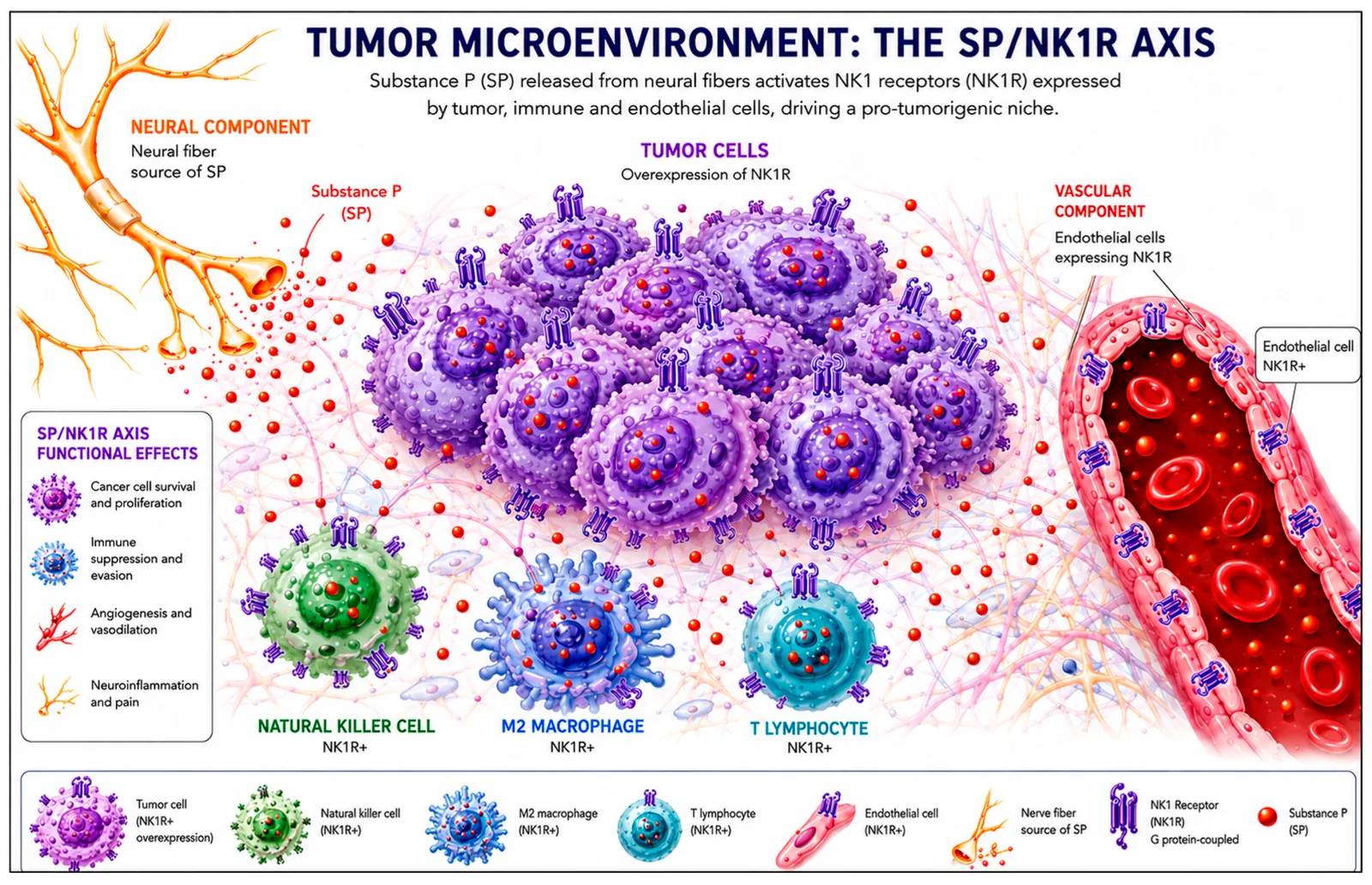
The cellular components of the TME—cancer cells, immune cells (macrophages, NK cells, lymphocytes), and endothelial cells—express SP and NK-1R. SP is also found in the extracellular spaces and in the bloodstream. Neural components also express SP and NK-1R. All components release SP.

The TME is a complex ecosystem that encompasses and surrounds the tumor. The TME plays a crucial role in in cancer progression, metastasis, immune evasion, and treatment resistance [118]. The TME has three components (Figure 1):

(A) The tumor mass (composed of tumor cells): SP and NK-1R are located in the cytoplasm and nucleus of tumor cells [9,39] (Table 1), suggesting potential autocrine, paracrine, intracrine, and/or endocrine functions [60]. Autocrine function: Cancer cells synthesize and release SP into the extracellular space after binding to NK-1R receptors of the tumor cell itself, producing both mitogenic and anti-apoptotic effects in the tumor cells. Paracrine function: Tumor cells synthesize and release SP into the extracellular space. SP binds to NK-1R located on other tumor cells and endothelial cells, increasing mitogenesis in tumor and endothelial cells (angiogenesis), as well as exerting an anti-apoptotic effect in tumor cells [113]. Furthermore, SP binds to NK-1R on tumor cells and inflammatory cells, stimulating the secretion of cytokines, including IL-1β, IL-6, IL-8, TNF-α, GM-CSF, and leukemia inhibitory factor (LIF) [8,119].
(B) The stroma in turn has two components: one cellular and one non-cellular. Cellular components: immune cells (T cells, B cells, macrophages and NK cells), stromal cells composed mainly of cancer-associated fibroblasts (CAFs) and vascular cells (endothelial cells). SP and NK-1R are found in the cellular components of the TME. NK-1R-T isoform is present in several immune cell populations, including monocytes, natural killer (NK) cells, and T lymphocytes. Its functional sensitivity to SP, however, differs from that of the NK-1R-F. Whereas NK-1R-F-expressing normal cells can respond to SP at nanomolar concentrations, activation of NK-1R-T in monocytes and macrophages generally requires higher, micromolar concentrations of the peptide [36,37]. Tumor development and the TME can increase local and systemic SP availability, with malignant tissues contributing to an endocrine-like source of this neuropeptide. Accordingly, circulating SP concentrations have been reported to be higher in individuals with cancer than in healthy subjects [28,29,30,31]. Increased systemic SP levels are associated with enhanced inflammatory activity, whereas pharmacological inhibition of NK-1R can attenuate inflammation and cytokine production in vivo [36,37]. Under physiological conditions, the predominance of NK-1R-T in immune cells may limit their activation in response to the relatively low concentrations of SP present in normal tissues, thereby contributing to immune homeostasis. In contrast, within the SP-rich environment of a tumor, increased peptide concentrations may reach the threshold required for NK-1R-T activation, promoting immune-cell responses and influencing monocyte/macrophage polarization [37]. Furthermore, perineural invasion (PNI) generates a TME that includes the overexpression of fibroblasts, macrophages, and angiogenesis, which could be responsible for neuropathic pain. In the TME, dormant fibroblasts are activated, becoming CAFs [120], and CAFs overexpress NK-1R in membrane and SP [47]. Non-cellular components: ECM and signaling molecules, cytokines, chemokines, growth factors and hormones. SP also modulates all these components of TME [60].
(C) Nervous system: sensory nerve fibers, neurotransmitters and neuropeptides. SP/NK-1R is known to be expressed in the nervous system and to release SP from sensory fibers in different tissues [8,121]. Taken together, the SP/NK-1R system acts as a mediator of the onconeuroimmune crosstalk at the interface between neural, immune and cancer signaling within the TME. SP is released in cancer not only from sensory fibers innervating the tumor but also directly from tumor cells and immune cells, thereby establishing autocrine and paracrine signaling circuits that promote tumor progression. Acting through its receptors, SP exerts multiple effects on both tumor and immune cells and sensory neurons. Increased SP levels have been associated with greater tumor cell proliferation and angiogenesis. Furthermore, high concentrations of SP promote macrophage polarization toward an M2 immunosuppressive phenotype [122], suppress NK cell cytotoxicity [123], and attenuate T cell activation [124], promoting a tolerogenic and immunosuppressive TME. In summary, SP exhibits oncogenic, neuronal, and immunological effects, highlighting its triple function as a tumor regulator, neuromodulator, and immunoregulator in cancer. Therefore, using NK-1R receptor antagonists, such as the drug aprepitant, modulation of the NK-1R/SP system could offer new opportunities for treating the onconeuroimmune components of tumors.

## 8. NK-1R Antagonist Aprepitant as a Broad-Spectrum Antitumor Drug in Pediatric Cancer

Non-peptide antagonists of NK-1R (compounds of different chemical composition but with similar stereochemical characteristics) are small, lipophilic molecules (that penetrate the brain) that are not degraded by peptidases. NK-1R antagonists are selective for NK-1R and counteract the pathophysiological functions of SP and act in a concentration- and time-dependent manner [9]. Numerous NK-1R antagonist compounds have been described; however, only four NK-1R antagonists are approved for clinical use in humans: aprepitant (and its intravenous prodrug fosaprepitant), rolapitant, netupitant and fosnetupitant [125].

### 8.1. NK-1R Antagonists Have Antiproliferative and Proapoptotic Effects in Pediatric Cancer Cells

A number of NK-1R antagonists, including L-733,060, L-732,138, CP 96-345, and aprepitant, have demonstrated antitumor activity in experimental models of pediatric malignancies [9]. Their effects have been investigated across several childhood cancers, including neuroblastoma, hepatoblastoma, retinoblastoma, osteosarcoma, rhabdoid tumors, B- and T-cell acute lymphoblastic leukemia (ALL), and acute myeloid leukemia (AML) [19]. Across these models, NK-1R blockade generally reduces tumor-cell proliferation and promotes cell death in a concentration-dependent manner [10,11,12,13,14,15,16,17,18]. In hepatoblastoma cells, both L-733,060 and aprepitant increase the proportion of apoptotic cells and are associated with caspase-3 activation and poly(ADP-ribose) polymerase (PARP) cleavage [14]. Similarly, L-733,060-mediated inhibition of NK-1R in glioma cells suppresses basal Akt kinase activity and promotes apoptosis, accompanied by caspase-3 cleavage and PARP proteolysis [68]. Taken together, these preclinical findings support further investigation of NK-1R antagonists, particularly aprepitant, as potential therapeutic agents for pediatric cancers.

### 8.2. NK-1R Antagonists Have Anti-Warburg Effect in Pediatric Cancer

Cancer cells exhibit a distinctive metabolic phenotype known as the Warburg effect, in which glucose is preferentially metabolized via glycolysis and converted to lactate in the presence of oxygen [126]. This metabolic reprogramming supports the high biosynthetic demands associated with uncontrolled tumor growth. In addition to generating ATP, the increased glycolytic activity provides the metabolites necessary for the synthesis of essential cellular components, such as nucleic acids, lipids, and proteins. Furthermore, key glycolytic intermediates, such as glucose-6-phosphate, dihydroxyacetone phosphate, 3-phosphoglycerate, phosphoenolpyruvate, and pyruvate, can be redirected toward anabolic pathways that support the generation of new cells. Thus, the preference of malignant cells for aerobic glycolysis may reflect an adaptation that allows them to couple energy production with the increased biosynthetic requirements of rapid proliferation [127].

SP may contribute to the metabolic adaptation of NK-1R-expressing glioma cells by promoting glycogen mobilization and elevating intracellular Ca^2+^ levels [128]. These responses are abolished in a concentration-dependent manner by the selective NK-1R antagonist CP-96,345, indicating that they are mediated through NK-1R signaling [128]. Since glucose availability is essential for sustained glycolytic metabolism, interference with this pathway could deprive tumor cells of a critical metabolic substrate and potentially compromise their survival. Furthermore, evidence suggests that glycolytic activity in malignant cells is associated with NK-1R abundance, and tumors exhibiting higher receptor expression also show increased glycolytic rates [113]. GSK-3α/β is an additional component of this regulatory mechanism. In glioma cells, SP-mediated NK-1R activation promotes phosphorylation and functional inhibition of GSK-3α/β, thereby favoring glucose production [68]. Furthermore, GSK-3 inhibitors are known to reduce glucose production and increase glycogen synthesis from L-glucose. Therefore, they could decrease glucose concentrations in hepatoma cells [129]. Similarly, NK-1R antagonists reduce GSK-3α/β phosphorylation in a concentration-dependent manner, limiting glucose generation and promoting glycogen synthesis, thus counteracting the metabolic phenotype associated with the Warburg effect [68]. This anti-Warburg therapeutic effect of NK-1R antagonist drugs is very important because few drugs specifically targeting tumor glycolysis have reached clinical practice, although several are under investigation.

These preclinical observations raise the possibility of targeting tumor glycolysis therapeutically, beyond its current diagnostic use in PET imaging, by treating cancer patients with NK-1R antagonists, such as the drug aprepitant.

### 8.3. NK-1R Antagonists Have Anti-Angiogenic Effects in Pediatric Cancer

Angiogenesis is a hallmark of cancer, since solid tumors require a blood supply to grow beyond a few millimeters. Angiogenesis has been associated with an overexpression of NK-1R. NK-1R has been reported to be found in the intra- and peritumor blood vessels in a large majority of tumors [45]. Moreover, SP, after binding to NK-1R of endothelial cells, can directly stimulate the process of angiogenesis, through induction of endothelial cell proliferation. In contrast, two peptides, NK-1R antagonists, counteract SP-induced angiogenesis [32]. In osteosarcoma cells, aprepitant decreases the expression of VEGF [18]. In hepatoblastoma xenografts, aprepitant reduced both tumor volume and the vascularized tumor area [14].

### 8.4. NK-1R Antagonists Exert Anti-Metastatic Effect in Pediatric Cancer

The migration of tumor cells is a fundamental step in the development of local invasion and metastatic disease, with metastatic dissemination being responsible for a substantial proportion of cancer-related mortality [5]. In pediatric malignancies, the ability of tumor cells to invade surrounding tissues and spread to distant organs is a major determinant of poor clinical outcomes. Metastatic disease may arise during treatment of the primary tumor, shortly thereafter, or even years following completion of therapy. This complex process involves multiple sequential events, including epithelial–mesenchymal transition (EMT), disruption of intercellular adhesion, extracellular matrix degradation through the release of matrix metalloproteinases (MMPs), acquisition of migratory properties, intravasation into the circulation, survival and transport within the bloodstream, and subsequent extravasation into distant tissues. Inflammatory changes and increased vascular permeability can facilitate both intravasation and extravasation. It has been reported that SP signaling participates in several of these steps [60]. EMT is a dynamic cellular program through which epithelial cells lose their characteristic polarity and cell–cell contacts and acquire mesenchymal features, including enhanced motility and invasive capacity, increased resistance to apoptosis, and alterations in extracellular matrix (ECM) production [118]. Consequently, EMT can facilitate tumor-cell migration and invasion while promoting dissemination to distant organs and increasing resistance to conventional chemotherapy and radiotherapy [60].

**Figure 2:**
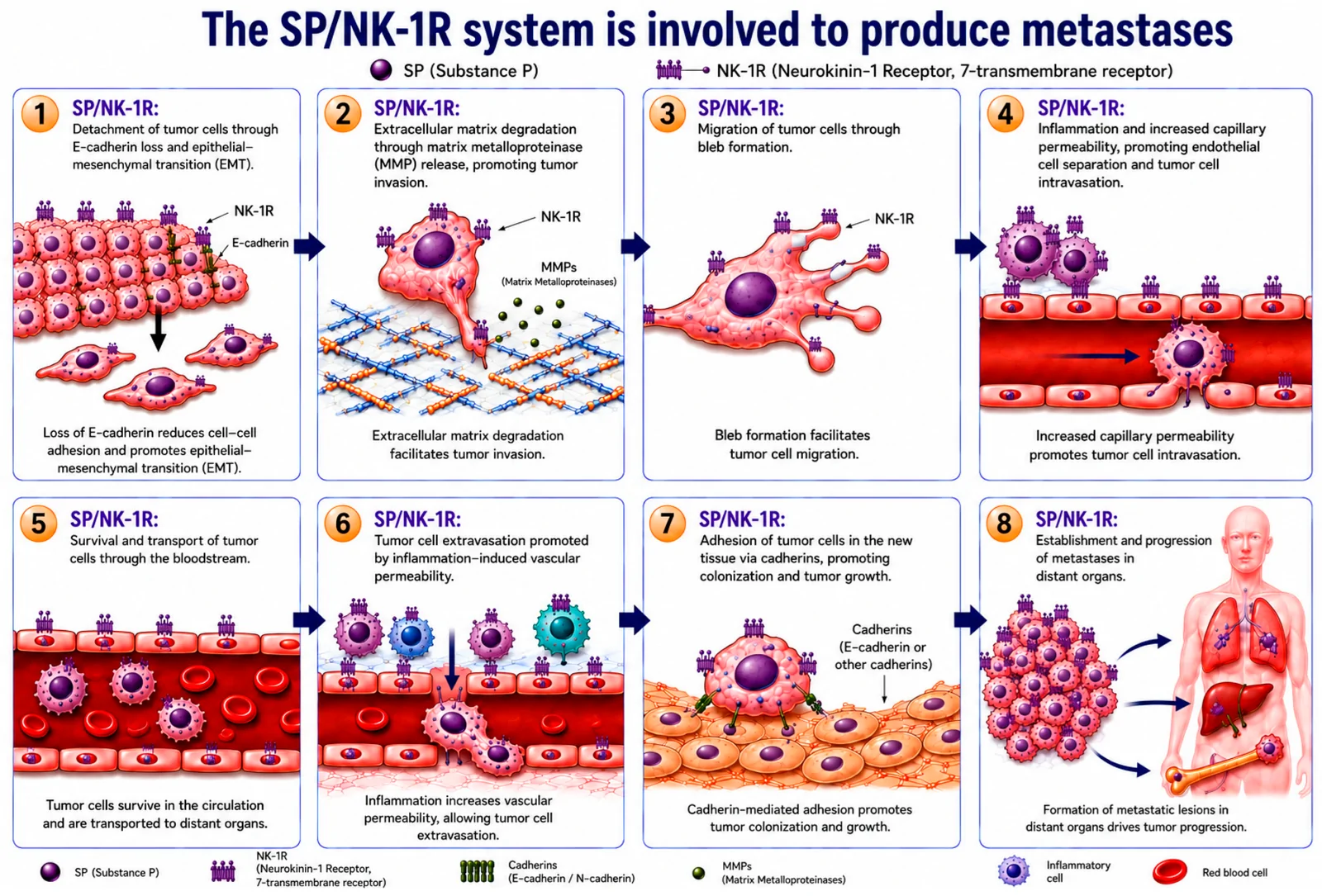
Substance P (SP) participates in the successive steps of the metastatic cascade.

**Figure 3:**
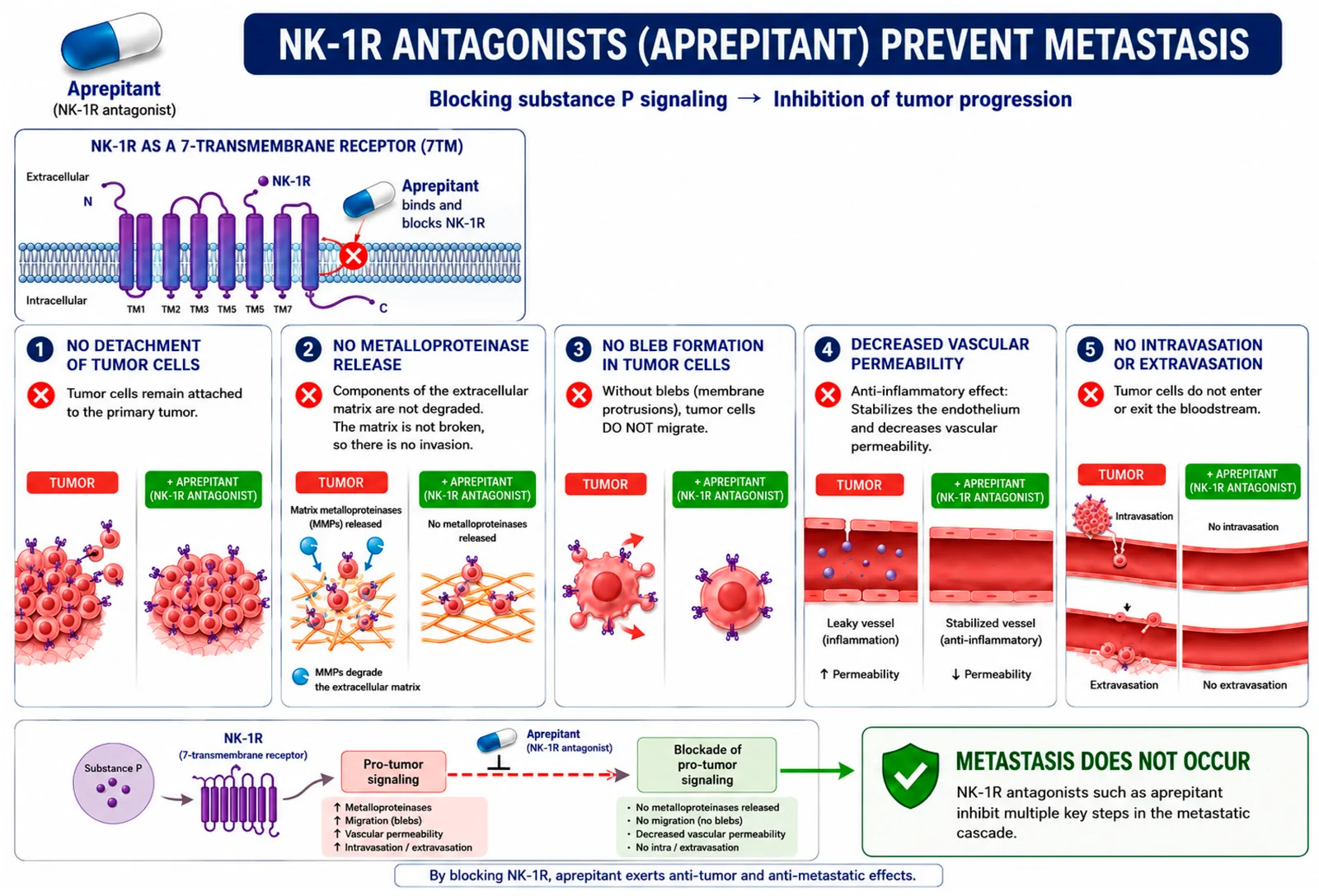
The therapeutic use of aprepitant prevents all steps of SP-mediated metastasis.

SP has been involved in several mechanisms that facilitate tumor invasion and metastatic dissemination. In head and neck cancer cells, SP promotes EMT and increases the expression of MMPs, thereby enhancing invasive behavior [130] (Figure 2). Conversely, pharmacological inhibition of NK-1R with L-703,606 reverses the expression of several EMT-related genes and produces antimetastatic effects [130] (Figure 3). Similarly, aprepitant has been shown to attenuate EMT progression in a Balb/C mouse model of endometriosis [121] (Figure 3). MMP activity also contributes to the development of tumor-associated neovasculature, facilitating intravasation and metastatic dissemination [131]. Through NK-1R activation, SP increases MMP-9 and VEGF-C expression, two factors associated with vascular invasion and lymphatic metastatic spread, respectively. In endometrial adenocarcinoma cells, treatment with the NK-1R antagonist L-733,060 suppresses the SP-induced upregulation of both proteins and consequently reduces invasive and metastatic potential [132]. In OS, aprepitant has likewise been reported to decrease NF-κB expression and downstream targets such as MMP-2, MMP-9, and VEGF-A [18] (Figure 3). At an IC_50_ of approximately 31.55 μM, corresponding to an in vivo dose of approximately 15 mg/kg/day, aprepitant reduced the invasive and metastatic behavior of OS cells [18]. In addition to these molecular mechanisms, membrane blebbing represents an important component of cancer-cell motility, particularly under conditions of reduced adhesion to the extracellular substrate [133]. SP–NK-1R signaling can induce morphological changes in malignant cells, including the formation of membrane blebs, which facilitate cellular movement, tissue infiltration, and dissemination [134] (Figure 3). Notably, SP induces a more rapid and transient blebbing response in U373MG glioma cells than in non-malignant cells, further supporting a role for this pathway in tumor-cell migration and invasion [135].

Although biopsy and surgical resection are essential for the diagnosis and treatment of solid tumors, surgical manipulation can, under certain circumstances, contribute to the dissemination of tumor cells [136,137,138]. Both tissue injury and surgical intervention can trigger neurogenic inflammation and pain, processes in which SP is an important mediator [8,120,139]. Since SP/NK-1R signaling has been associated with tumor cell proliferation, invasion, and metastasis, the inflammatory response induced by these procedures can potentially create conditions that favor tumor progression. This consideration may be particularly relevant in pediatric malignancies, in which NK-1R is frequently overexpressed and circulating SP levels may be elevated compared to healthy individuals [28,29,30,31]. Consequently, research into strategies aimed at limiting treatment-associated mechanisms that could contribute to recurrence or metastatic spread is warranted. One potential approach is perioperative inhibition of NK-1R with aprepitant (Figure 3) [140]. Preclinical evidence supporting NK-1R as a therapeutic target has shown that its inhibition can interfere with the mechanisms involved in tumor invasion and metastasis. In OS, exposure to aprepitant, corresponding to an extrapolated IC_50_ of approximately 15 mg/kg, has shown antimetastatic activity, raising the possibility of exploring aprepitant administration prior to biopsy or surgical resection as a strategy to reduce the risk of metastasis. However, this proposed perioperative use remains under investigation and requires validation in appropriate preclinical and clinical studies before it can be considered a preventive treatment for pediatric cancer metastasis. Further evidence supports a role for the SP/NK-1R axis in tumor cell dissemination. In NB, increased *TAC1* expression has been linked to increased generation of circulating tumor cells (CTCs). Mechanistically, TWIST1 directly promotes the transcription of *TAC1*, which encodes SP, thereby activating the *TAC1–TACR1* signaling pathway and facilitating communication between NB cells and tumor-associated endothelium. This interaction promotes vascular remodeling through endothelial senescence and disruption of intercellular tight junctions, ultimately favoring metastatic spread. Notably, aprepitant was able to inhibit *TACR1*-dependent endothelial senescence and reduce metastasis in vivo, highlighting the potential therapeutic relevance of NK-1R blockade in metastatic NB [54] (Figure 3). Consistent with these observations, a recent study on pancreatic cancer found that increased cytoplasmic expression of the NK-1R in tumor samples was associated with greater intraoperative release of CTCs and mobilization of CTC clusters. Furthermore, increased cytoplasmic expression of the NK-1R and elevated SP levels were associated with reduced metastasis-free survival and reduced overall survival, respectively [140]. Taken together, these findings warrant investigation of perioperative NK-1R inhibition with aprepitant as a potential strategy to limit tumor cell dissemination and metastatic progression associated with surgery in pediatric cancer.

### 8.5. NK-1R Antagonists Have Both Anti-Inflammatory and Immunomodulatory Effects

SP is a key mediator of neurogenic inflammation and exerts its effects primarily through interaction with the NK-1R, which is expressed on various inflammatory and endothelial cell populations [8]. Activation of this receptor contributes to the development of inflammatory responses and pain associated with tissue damage. Conversely, pharmacological blockade of NK-1R with antagonists such as aprepitant attenuates SP-mediated inflammatory effects in a concentration-dependent manner, resulting in an overall anti-inflammatory response. Furthermore, SP/NK-1R signaling is involved in inflammatory pain through its actions on specialized sensory neurons involved in the detection and transmission of noxious stimuli.

The potential analgesic and anti-inflammatory properties of aprepitant have been investigated using lipopolysaccharide (LPS)-activated BV-2 microglial cells and corresponding animal models of inflammatory pain. In these experimental systems, aprepitant reduced neuronal excitability in dorsal root ganglion neurons and attenuated microglial activation. These effects were accompanied by decreased JNK and p38 MAPK phosphorylation and reduced transcriptional and protein expression of several pro-inflammatory mediators, including MCP-1, TNF-α, IL-6, and IL-1β. In BV-2 cells, aprepitant also decreased LPS-induced nuclear accumulation of the p65 subunit of NF-κB. Taken together, these findings indicate that aprepitant may relieve inflammatory pain in mice, at least in part, by inhibiting JNK/p38 MAPK activation and suppressing NF-κB-dependent inflammatory signaling [139].

SP also functions as an important neuroimmune regulator, and elevated circulating concentrations of this neuropeptide have been associated with alterations in the number and activity of natural killer (NK) cells. Since NK cells express NK-1R, SP can directly influence their functional responses through receptor activation. Increased plasma levels of SP have been correlated with a reduction in the number of circulating NK cells, as well as with impaired cytotoxic activity. Experimental evidence indicates that SP suppresses NK cell cytotoxicity and degranulation, effects that can be partially reversed by the selective NK-1R antagonist CP-96,345 [123]. This mechanism could, therefore, contribute to the compromised NK cell-mediated immune surveillance observed in cancer patients, particularly in the context of elevated plasma concentrations of SP [28,29,30,31]. Conversely, pharmacological inhibition of NK-1R with drugs such as aprepitant could represent a potential strategy to restore or improve NK cell function.

Macrophages are an important cellular component of the TME and exhibit considerable functional plasticity, ranging from classically activated M1 phenotypes, generally associated with antitumor activity, to alternatively activated M2 phenotypes, frequently linked to tumor support functions. SP has been associated with promoting the acquisition of an M2-like phenotype in inflammatory macrophages. In particular, SP can induce an alternatively activated phagocytic M2 phenotype (M2SP) in GM-CSF-derived proinflammatory macrophages. This process involves the activation of the PI3K/Akt/mTOR/kinase S6 signaling cascade and is accompanied by increased expression of characteristic M2-associated markers, such as arginase-1, CD163, and CD206. In contrast, pharmacological blockade of NK-1R with RP67580 prevents SP-induced macrophage polarization, thus supporting the function of the SP/NK-1R pathway in the tumor-associated immune microenvironment [122].

The immune system is involved in the control of cancer progression, whereas malignant cells can exploit immune-regulatory mechanisms to evade immune surveillance. Programmed cell death protein 1 (PD-1) is an immune checkpoint receptor expressed on the surface of immune effector cells and is predominantly engaged by its ligand, PD-L1. The PD-1/PD-L1 signaling axis is essential for maintaining peripheral T-cell tolerance and controlling inflammatory responses. However, in the context of cancer, aberrant or increased PD-L1 expression represents an important mechanism by which tumor cells evade immune surveillance. Accordingly, extensive evidence has shown that therapeutic inhibition of either PD-1 or PD-L1 using specific monoclonal antibodies can restore antitumor immune activity and produce beneficial therapeutic effects [141]. Interestingly, treatment with aprepitant in HIV-1-infected adults was associated with reduced circulating SP concentrations and a decrease in the population of CD4^+^ PD-1-positive cells [124]. These findings raise the possibility that NK-1R inhibition with aprepitant could modulate SP-associated immune suppression and potentially interfere with mechanisms contributing to tumor immune evasion.

## 9. Combination Therapy of Chemotherapy Plus Aprepitant

The drug aprepitant is used for CINV caused by chemotherapy drugs such as cisplatin, doxorubicin, etc. An important factor to consider for the clinical use of aprepitant is a substrate and a moderate inhibitor of CYP3A4, and an inducer of CYP2C9; it may therefore increase the exposure to co-administered cytotoxic agents metabolized by CYP3A4, so it can lead to drug interactions, especially with chemotherapy agents that are metabolized by this pathway. If the concentration of the cytostatic agent increases as a result of the interaction, the clinical consequences may be serious, since these drugs already produce significant toxicity at therapeutic doses. For information on the use of aprepitant in combination with various chemotherapy drugs, we recommend visiting the website https://www.drugs.com/drug_interactions.html (accessed on 6 September 2026).

It has been reported that combination therapy of chemotherapy and aprepitant displays chemosensitization in tumor cells [25,26,27], counteracting the serious side effects of chemotherapy by producing organ protection (cardioprotection, nephroprotection, hepatoprotection, neuroprotection and testicular protection) and cytoprotection [23,24,25,27,142,143,144,145]. Infertility after chemotherapy is a common side effect in pediatric cancer. Cisplatin and cyclophosphamide are antineoplastic drugs used to treat various types of pediatric cancer. Unfortunately, they can cause testicular damage, leading to long-term infertility and irreversible azoospermia. In this regard, the combination of aprepitant with cisplatin or cyclophosphamide in mice shows effective protection against cisplatin and cyclophosphamide-induced testicular toxicity [144,145]. Furthermore, preclinical evidence indicates that NK-1R blockade may enhance the efficacy of conventional chemotherapy in osteosarcoma (OS). In an in vitro model using MG63.2 OS cells, the addition of the NK-1R antagonist L-733,060 to commonly used chemotherapeutic agents, including doxorubicin, cisplatin, and ifosfamide, potentiated their antitumor effects. These findings suggest that NK-1R inhibition may have a chemosensitizing role and could potentially improve the response of OS cells to standard chemotherapy [15]. However, when non-tumor human HEK-293 cells were treated with an NK-1R antagonist, L-733,060, prior to exposure to chemotherapy drugs, they were partially protected from the cytotoxicity of chemotherapy [15]. In addition, the combination of aprepitant and cisplatin or doxorubicin against HB cells increases the anticancer activity of these chemotherapy drugs [14].

Based on the above, the use of NK-1R antagonists has been proposed as a new strategy to overcome cancer resistance [146]. One of the mechanisms by which the combination of chemotherapy drugs plus NK-1R antagonists, such as aprepitant, can overcome cancer resistance is counteracting the Warburg effect [126]. Since NK-1R antagonists prevent the breakdown of glycogen into glucose in tumor cells [128], the lack of glucose leads to a lack of energy and weakens tumor cells, making them more sensitive to chemotherapy. Taken together, the findings described above indicate that combining aprepitant with conventional chemotherapy may increase the sensitivity of pediatric cancer cells to cytotoxic treatment. This potential chemosensitizing effect warrants further investigation of aprepitant as an adjunctive therapeutic strategy in pediatric cancer.

## 10. Clinical Experience with Aprepitant and Safety

Aprepitant was introduced into clinical practice after its approval by the U.S. Food and Drug Administration (FDA) in March 2003 as the first neurokinin-1 receptor (NK-1R) antagonist indicated for the prevention of chemotherapy-induced nausea and vomiting (CINV) in adults. Its use was subsequently extended to the prevention of postoperative nausea and vomiting in adults in June 2006 and received pediatric approval in 2015 [147]. The recommended regimen consists of 125 mg on the first day followed by 80 mg on days 2 and 3 in adults, whereas pediatric patients receive weight-adjusted doses of 3 mg/kg on day 1 and 2 mg/kg on days 2 and 3, subject to the corresponding adult maximum dose. Aprepitant is generally well tolerated, with commonly reported adverse effects including constipation, fatigue, headache, decreased appetite, hiccups, and diarrhea [148]. Compared with conventional cytotoxic chemotherapy, which has a relatively narrow therapeutic window and can produce substantial systemic toxicity at effective doses, aprepitant has demonstrated a comparatively favorable safety profile across multiple clinical and experimental studies.

Evidence from randomized clinical trials generally supports the use of aprepitant as part of antiemetic therapy without a clear increase in hematological toxicity. A systematic review and meta-analysis including 451 patients found that a three-drug antiemetic regimen containing aprepitant significantly reduced the occurrence of chemotherapy-induced nausea and vomiting (CINV) without increasing the risk of febrile neutropenia [149]. Nevertheless, individual studies in pediatric patients with bone tumors have reported relatively high rates of febrile neutropenia among those receiving aprepitant, with one study reporting an incidence exceeding 43.8 cases per 100 patients [150]. Conversely, a larger meta-analysis comprising 23 randomized controlled trials and 7956 participants found no statistically significant association between aprepitant administration and the occurrence of febrile neutropenia, supporting its overall safety in the populations evaluated [151]. Furthermore, consistent with the previous study, the cytotoxicity of aprepitant on lymphocytes is known to have an IC_50_ of 176 μM [17]; if we convert that to mg/kg, it would be approximately 88 mg/kg, or about 6000 mg per day for a 70 kg adult. Because neutropenia generally results from impaired bone marrow function, the absence of clinically relevant myelotoxicity associated with aprepitant would not, in principle, support a direct causal relationship between this drug and febrile neutropenia in patients with cancer, including children.

Carcinogenicity studies of aprepitant have been conducted in rodent models. In these experiments, tumor development was associated with species-specific hepatic CYP-mediated metabolism and occurred at relatively high exposure levels. Carcinogenicity studies were conducted in mice and rats for 2 years with oral aprepitant. Reported neoplastic findings in mice included skin fibrosarcomas at doses of 125–500 mg/kg/day, hepatocellular adenomas and carcinomas at 1000–2000 mg/kg/day, thyroid parafollicular cell carcinomas at 1000 mg/kg twice daily. Rats developed hepatocellular carcinomas at doses of 125 to 1000 mg/kg twice daily and follicular thyroid cell carcinomas at the same dose [9].

It is important to note that, for the possible treatment of cancer patients, the extrapolated dose of aprepitant (20 mg/kg/day) [9,147] would be lower than the carcinogenic doses mentioned above. Conversely, aprepitant inhibits the mitogenesis of tumor cells and cancer cells die by apoptosis, thus counteracting the mitogenic action of SP [9,19].

In humans, few studies have been carried out on aprepitant at high doses:

(1) A case report described the compassionate use of aprepitant at a dose of 1140 mg/day for 45 days in combination with palliative radiotherapy in a patient with lung cancer with a tumor mass (8 × 7 cm) in the right hemithorax. Six months after completion of the treatment, complete disappearance of the tumor mass was reported. The clinical course was accompanied by a favorable safety profile, with no severe adverse effects attributed to the treatment. The patient remained in good general condition, gained weight, and maintained normal biochemical and hematological parameters. Additionally, no significant toxicity related to the radiotherapy was documented [152].
(2) In a clinical trial involving patients with moderate-to-severe depression, administration of 300 mg/day for 30 days was considered safe and well tolerated, with adverse events comparable to those observed with placebo and an antidepressant effect similar to that of paroxetine [20].
(3) Likewise, a two-week course of aprepitant at 375 mg/day in individuals with HIV infection was well tolerated and was associated with reductions in circulating SP levels, CD4^+^ PD-1-positive cells, and soluble CD163 concentrations. These findings suggest that NK-1R blockade may exert both anti-inflammatory effects and potentially attenuate SP-associated mechanisms of immune evasion [124].

More recently, data from a Norwegian national registry including 13,811 women diagnosed with early-stage breast cancer between 2008 and 2020 were analyzed to examine subsequent metastatic disease and mortality. During follow-up through the end of 2021, patients who received aprepitant as part of their antiemetic regimen alongside chemotherapy were reported to have a lower risk of metastasis and death. Notably, the aprepitant regimen evaluated corresponded to the standard CINV schedule of 125 mg on day 1 followed by 80 mg on days 2 and 3 [153]. In agreement with this study, another study conducted in a cohort of 654 patients highlighted the potential chemosensitizing and prognostic benefits of aprepitant in breast cancer patients, showing significantly improved survival in terms of overall survival (OS) and disease-free interval (DFI) in the TNBC, HR-positive and HER2-positive breast cancer subtypes, and a reduction in recurrences [154]. Therefore, the use of aprepitant in combination with chemotherapy has both therapeutic, anti-CINV effects and enhances the antitumor activity of chemotherapy drugs.

## 11. NK-1R Antagonist Aprepitant as an Intelligent Bullet in Pediatric Cancer Therapy

The concept of the “magic bullet” was introduced by the German physician and Nobel laureate Paul Ehrlich in the early twentieth century. Ehrlich proposed that it should be possible to develop therapeutic agents capable of selectively targeting disease-causing organisms while sparing healthy tissues. He referred to this hypothetical principle as *Zauberkugel*, or “magic bullet,” emphasizing a treatment that would act preferentially on the pathological target rather than the host. This concept has subsequently influenced the development of molecularly targeted therapies. Within the context of contemporary oncology, NK-1R antagonists have emerged as potential targeted agents because of their ability to interfere with SP/NK-1R signaling in tumor cells while exhibiting a comparatively favorable safety profile. Consequently, these compounds have been proposed as potential “intelligent bullets” or modern equivalents of Ehrlich’s magic bullet for cancer treatment [9,19]. This concept goes even further, since they not only attack invasive tumor cells and do not produce serious side effects, but they also have beneficial therapeutic effects for the patient, such as CINV effects, antidepressant effects, anti-inflammatory effects, counteracting immune evasion, hepatoprotection, neuroprotection, cardioprotection, nephroprotection and protection against testicular toxicity [7,20,21,22,23,24,25,139,144,145].

In addition, aprepitant could be considered an intelligent bullet in pediatric cancer therapy. NK-1R antagonists are different drugs from chemotherapy, since the latter (at therapeutic doses) produces serious side effects, while NK-1R antagonists, such as the drug aprepitant (at therapeutic doses), is safe and well tolerated, and can exert numerous therapeutic effects. In addition, the NK-1R antagonist aprepitant, in combination therapy with chemotherapy or radiotherapy, produces chemosensitization and radiosensitization of cancer cells and counteracts the severe side effects of chemotherapy and radiotherapy [25]. The data mentioned above indicate that NK-1R could be a novel therapeutic target and that aprepitant could be a new drug for the treatment of pediatric cancer. The therapeutic dose of aprepitant for the treatment of pediatric cancer has been suggested to be 20 mg/kg/day [9].

## 12. Conclusions

Taken together, the data reported above demonstrate that NK-1R may be a potential new therapeutic target for the treatment of pediatric cancer, since pediatric cancer, tumor and cancer cells overexpress SP and NK-1R. NK-1R is essential for the viability of pediatric cancer cells; however, it is not essential for normal non-tumor cells. SP, after binding to NK-1R of pediatric cancer cells, elicits proliferation and antiapoptotic effect in pediatric cancer cells, and also induces the Warburg effect. SP also stimulates the proliferation of endothelial cells and angiogenesis in the tumor and induces invasion and migration in tumor cells for metastasis.

In contrast, treatment with NK-1R antagonists, such as the drug aprepitant, exerts antitumor activity (antiproliferative, proapoptotic, anti-Warburg effect, antiangiogenic, antimetastatic, and tumor-reducing) in pediatric cancer.

The drug aprepitant, an NK-1R antagonist, and similar drugs, exert a concentration- and time-dependent antitumor activity. In addition, they are broad-spectrum antitumor drugs effective against all types of pediatric cancer studied.

(1) They may be useful in treatments for brain tumors, such as pediatric glioma.
(2) They could prevent cancer metastasis.
(3) They have an anti-inflammatory effect and counteract immune evasion.
(4) In combination therapy with chemotherapy or radiotherapy, they have two important therapeutic effects: they produce tumor chemosensitization or radiosensitization (they overcome cancer resistance) and counteract the serious side effects of chemotherapy or radiotherapy.
(5) At high doses they are safe and well tolerated, and have no serious side effects; in addition, it is a drug that has anti-CINV properties.

Therefore, it is necessary to repurpose aprepitant as an antitumor drug, in combination with chemotherapy or radiotherapy in pediatric cancer, and phase I and II clinical trials are urgently needed to evaluate its safety and efficacy in treating pediatric cancer patients.

## Data Availability

No new data were created or analyzed in this study. Data sharing is not applicable to this article.
